# Isolation of Biotype 1 Serotype 12 and Detection of *Actinobacillus pleuropneumoniae* from Wild Boars

**DOI:** 10.3390/pathogens11050505

**Published:** 2022-04-24

**Authors:** Rita Sárközi, László Makrai, László Fodor

**Affiliations:** Department of Microbiology and Infectious Diseases, University of Veterinary Medicine, P.O. Box 22, H-1581 Budapest, Hungary; rita.sarkozi@gmail.com (R.S.); makrai.laszlo@univet.hu (L.M.)

**Keywords:** *Actinobacillus pleuropneumoniae*, wild boar, PCR, isolation

## Abstract

*Actinobacillus pleuropneumoniae* is a major pathogen of swine, which can cause severe pleuropneumonia in pigs, but sometimes the disease can be generalized. Diseases caused by *A. pleuropneumoniae* are frequent all over the world, resulting in high losses among domestic pigs. However, our knowledge on the occurrence of *A. pleuropneumoniae* in wild boars and feral pigs is limited. We aimed to examine the carriage of *A. pleuropneumoniae* by hunted wild boars. The presence of *A. pleuropneumoniae* was examined in tonsils of 68 hunted wild boars collected at a game processing unit. An in-house designed species-specific PCR test was used to detect the gene of Apx IV toxin, and the samples were inoculated on a modified selective agar. *A. pleuropneumoniae* was detected in 10 animals (14.7%) by PCR and one *A. pleuropneumoniae* serotype 12 strain was isolated. The antibiotic resistance pattern of the strain resembled field strains that were isolated from farmed pigs in Hungary. This is the first case for the detection of *A. pleuropneumoniae* not only using PCR or ELISA, but also its isolation, identification, and serotyping.

## 1. Introduction

*Actinobacillus pleuropneumoniae* is a major pathogen of swine. There are different virulence variants of the agent, the highly virulent ones can cause severe fibrino-haemorrhagic, necrotic pneumonia, and fibrinous pleuritis in pigs, but sometimes the disease can be generalized in the infected animals [1,2]. The moderately virulent strains play an important role in the aetiology of porcine respiratory disease complex [3]. The host range of *A. pleuropneumoniae* is very narrow, it can cause disease only in swine (*Sus scrofa*), including domestic pigs, wild boars, and feral pigs. However, it was identified once as a causative agent in laying hens, showing clinical signs of infectious coryza [4]. *A. pleuropneumoniae* is carried in tonsils by infected animals. In addition, it is shed in respiratory discharge, especially when the animals are coughing [2,5,6].

*Actinobacillus pleuropneumoniae* has two biotypes, biotype I strains can replicate only in the presence of nicotinamide adenine dinucleotide (NAD, V-factor), which is different from the biotype II strains that do not need it. Virulence of *A. pleuropneumoniae* depends on several virulence factors, including four types of toxins, fimbria, outer membrane proteins, ability of biofilm formation, presence of transporter systems, and different enzymes, that contribute to the virulence of the agent [7,8,9,10]. To date, a total of 19 serovars have been identified on the basis of surface soluble capsular polysaccharide antigens [11]. The frequency of different biotypes and serovars of *A. pleuropneumoniae* shows great geographical differences [2].

Diseases caused by *A. pleuropneumoniae* are frequent all over the world, and can cause high losses among domesticated pigs. However, our knowledge on the occurrence of *A. pleuropneumoniae* in wild boars and feral pigs is limited. A large part (52%) of hunted wild boars proved to be seropositive for *A. pleuropneumoniae* in Slovenia [12]. More than two thirds (69.7%) of the serum samples were positive in the United States, and the presence of antibodies against different serotypes of *A. pleuropneumoniae* was identified [13]. In Finland 12.6%, while in Greece 90.5% of the sampled farmed wild boars had antibodies against the agent [14,15]. Moreover, seropositivity to *A. pleuropneumoniae* was described in Canada. However, the serotypes found in wild boars and domestic pigs were different [16,17]. Polymerase chain reaction (PCR) detected *A. pleuropneumoniae* in lung and tonsil samples of 38.5% of wild boars in Germany [18], but feral pigs in Australia proved to be free from *A. pleuropneumoniae* when lungs were examined with PCR [19]. Domestic pigs, wild boars, and feral pigs belong to a single species (*Sus scrofa*), they can be reservoirs of different viruses and bacteria, and can infect each other [20].

There are several methods to detect *A. pleuropneumoniae* in tonsils. Isolation of the agent on selective media [21] is rather difficult due to the fact that several other bacteria can overgrow it. Different PCR methods [22,23] or isolation by immunomagnetic separation [24,25] are also available.

The aim of the present examination was an evaluation of the presence of *A. pleuropneumoniae* in hunted wild boars in Hungary.

## 2. Results

All of the 38 field bacterial isolates investigated and type strains of *A. pleuropneumoniae* strains provided a positive reaction in the PCR test that was used to detect the species-specific *Apx IV* gene of *A. pleuropneumoniae*, while *Streptococcus suis*, *Staphylococcus aureus* subsp. *aureus*, *Escherichia coli*, and *Proteus mirabilis* did not react.

*Actinobacillus pleuropneumoniae* was detected by PCR in tonsils of 10 out of 68 (14.7%) wild boars. The positive animals were shot in different geographical locations in Hungary. Neither the positive nor the negative animals had any postmortem lesions. Moreover, no acute or chronic lesions of *A. pleuropneumoniae* could be observed.

**Figure 1:**
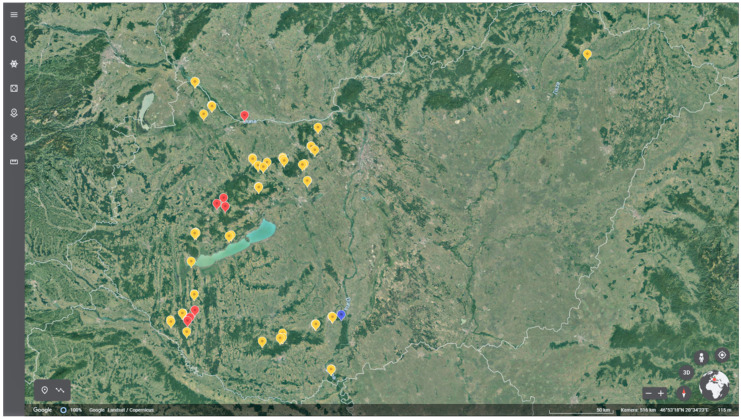
Detection of *A. pleuropneumoniae* from wild boars in Hungary (red: PCR positive; blue: PCR and culture positive; yellow: PCR negative).

Only one *A. pleuropneumoniae* strain could be isolated from tonsils of wild boars (1.47%), the same tonsil was also positive in the PCR test. The isolated *A. pleuropneumoniae* strain could be assigned to serotype 12. The results are shown on the map (Figure 1).

The minimum inhibitory concentrations of the examined antibiotics are presented in Table 1.

## 3. Discussion

The type specific in-house designed PCR, which was used to detect the gene of Apx IV toxin, proved to be reliable. All of the *A. pleuropneumoniae* type and field strains provided a positive reaction, while the reactions in the case of other bacterium species remained negative. Detection of the gene of Apx IV toxin is specific, only *A. pleuropneumoniae* strains carry it, while genes of Apx I-II-III can also be present in other species [10].

*Actinobacillus pleuropneumoniae* is a widespread bacterium in pig herds. However, there are limited data on the occurrence of the agent in wild boars and feral pigs. In Slovenia, the United States, and Canada, 52–100% of the animals were seropositive for *A. pleuropneumoniae* [12,13,16]. In addition, 12.6–90% of the farmed boars had antibodies against *A. pleuropneumoniae* [14,15]. *A. pleuropneumoniae* was detected in 38.5% of wild boars in Germany [18]. Only 14.7% of the wild boars carried *A. pleuropneumoniae* in tonsils in Hungary, but we could isolate an *A. pleuropneumoniae* strain from the tonsil of a wild boar. To the best of our knowledge, this is the first case for the isolation of the agent from wild boars. Isolation of *A. pleuropneumoniae* using selective media is not a simple task, since other, rapidly growing bacteria can overgrow it [2]. Moreover, isolation of certain serotypes of *A. pleuropneumoniae* is possible with immunomagnetic separation. However, selective isolation has to be used when the serotype of the targeted strain is not known [24,25].

The isolated strain could be assigned to serotype 12. Serotype 12 strains occur in Hungary, 3.3% of the *A. pleuropneumoniae* strains belong to this serotype [26].

There are two possible ways of infection among wild boars with *A. pleuropneumoniae*. Circulation of the agent within wild boar populations is an option. It is assumed in Canada, where wild boars were seropositive for serotype 14, which was not present in domestic pig populations [16]. The other option is infection from domestic pigs in the case of close contact. The *A. pleuropneumoniae* strain that we isolated was resistant against penicillin, gentamycin, spectinomycin, cefoperazone, tulathromycin, and tilmicosin. The antibiotic resistance pattern of the strain resembled the *A. pleuropneumoniae* strains isolated from farmed pigs. In addition, it was resistant against those antibiotics that are frequently used in Hungarian pig herds [27]. On the basis of these data, we could hypothesize that the wild boar was most probably infected from a farmed pig. Backyard-raised pigs are usual in rural areas of Hungary, where wild boar populations are also high.

Wild boars can infect farmed pigs with *A. pleuropneumoniae*. However, the risk is not high in the case of intensive units thanks to the high level of biosecurity, but they can infect backyard-raised pigs, especially during sow heat or estrus. The risk of infection is low in free ranging wild boars since close contact is needed for the infection, but it can be increased among farmed wild boars if the animal density is high.

## 4. Materials and Methods

### 4.1. Samples

Tonsils of 68 hunted wild boars were collected at a game processing unit in Hungary. With one exception the wild boars that were shot originated from 8 countries of the western part of the country. They were adult animals of both sexes in average body condition and seemed to be healthy. The tonsil samples were transported on ice to the laboratory immediately after collection, and were examined within 3 h.

### 4.2. Polymerase Chain Reaction

Polymerase chain reaction was used to detect *A. pleuropneumoniae* strains in tonsils. DNA was extracted from tonsils with QIAGEN QIAamp^®^ DNA Mini Kit (Qiagen Inc., Germantown, MD, USA), following the instructions of the producer. Our own PCR test was used for the identification of *A. pleuropneumoniae*. The primers (5′-ACG AACAAC GCG GCT AAT A-3′ and 5′-CTC ACC TAA CGG ACG AGT AAA-3′) were planned to detect the species-specific *Apx IV* gene of *A. pleuropneumoniae*. The reaction mixture contained 40.7 μL water, 5 μL buffer, 0.1 μL of 10 mM dNTP, 0.2 μL (5 U/μL) Taq polymerase, 2 μL template, and 1-1 μL of 10 μM primers. After 5 min of initialization at 94 °C, 35 cycles of denaturation at 94 °C, annealing at 55 °C, and extension at 72 °C were carried out, followed by a 7-min-long final elongation step. The amplicon was visualized by gel electrophoresis in 2% agarose gel in Tris–acetate–EDTA buffer (40 mM Tris–acetate and 1 mM EDTA pH 8.3) containing Green Nucleic Acid Gel Stain (Biocenter Ltd., Szeged, Hungary).

The specificity of the test was controlled with a single strain of each of the following: *S. suis*, *S. aureus* subsp. *aureus*, *E. coli*, and *P. mirabilis*, which are normally present in the pig, and 38 *A. pleuropneumoniae* strains including type and field strains. The strains were from our bacterium collection.

### 4.3. Bacterium Culture, Serotyping, Antibiotic Resistance

Tonsil samples were inoculated onto a selective agar medium, which was described by Jacobsen and Nielsen [21] and that we partially modified. Mueller–Hinton agar (Biolab Ltd., Budapest, Hungary) containing 5% defibrinated sheep blood was used rather than the meat and blood agar. In addition, 50 μg/mL NAD and 1% yeast extract (Biolab Ltd., Budapest, Hungary), as well as 100 μg/mL bacitracin, 1 μg/mL crystal violet, 50 μg/mL nystatin, and 1 μg/mL lincomycin (Sigma-Aldrich, St. Louise, MI, USA) were added. The agar plates were incubated at 37 °C for 72 h and read daily. The isolated bacteria were characterized on the basis of their morphological, cultural, and biochemical features [28,29]. In addition, the identification of the isolated *A. pleuropneumoniae* strain was confirmed with the above-mentioned PCR. The isolated *A. pleuropneumoniae* strain was serotyped in the indirect hemagglutination test using hyperimmune sera raised against 1–19 serotype strains of *A. pleuropneumoniae*, as described [26,30]. The antibiotic resistance of the isolated *A. pleuropneumoniae* strain was examined by measuring the minimum inhibition concentration in the microdilution method. Briefly, Mueller–Hinton broth (Biolab Ltd., Hungary) with the addition of 10 mg/mL Ca^++^ (CaCl_2_ × 2 H_2_O; Spektrum-3D Ltd., Debrecen, Hungary), 10 mg/mL Mg^++^ (MgCl_2_ × 6 H_2_O; Scharlab Hungary Ltd., Debrecen, Hungary) recommended for fastidious bacteria, and 67 μg/mL NAD medium were used. The tests were carried out in 96-well polystyrene microtiter plates (Kartell, Noviglio, Italy). The breakpoint values of *A. pleuropneumoniae* were derived, and when they were not available, the values of *Mannheimia haemolytica* and *Actinobacillus* sp. were used as reference [31,32].

## 5. Conclusions

*Actinobacillus pleuropneumoniae* was detected from tonsils of 14.7% of hunted wild boars, and one serotype 12 strain of *A. pleuropneumoniae* was isolated. This is the first case for the isolation of *A. pleuropneumoniae* from wild boars.

## Figures and Tables

**Table 1:** Antibiotic susceptibility of *Actinobacillus pleuropneumoniae* isolated from wild boars.

Antibiotic	MIC (μg/mL)	Susceptibility
Penicillin	1	Resistant
Amoxicillin	0.5	Susceptible
Amoxicillin–clavulanate	0.5	Susceptible
Ampicillin	0.125	Susceptible
Cefoperazone	16	Resistant
Gentamicin	16	Resistant
Spectinomycin	128	Resistant
Oxytetracycline	1	Intermediate susceptibility
Doxycycline	0.5	Susceptible
Tilmicosin	32	Resistant
Tiamulin	16	Susceptible
Chloramphenicol	2	Susceptible
Florfenicol	0.5	Susceptible
Enrofloxacin	0.06	Susceptible
Tulathromycin	128	Resistant

## Data Availability

Not applicable.
